# Serum Biomarkers Associated with Clinical Outcomes Fail to Predict Brain Metastases in Patients with Stage IV Non-Small Cell Lung Cancers

**DOI:** 10.1371/journal.pone.0146063

**Published:** 2016-01-05

**Authors:** Bob T. Li, Emil Lou, Meier Hsu, Helena A. Yu, Jarushka Naidoo, Marjorie G. Zauderer, Camelia Sima, Melissa L. Johnson, Mariza Daras, Lisa M. DeAngelis, Martin Fleisher, Mark G. Kris, Christopher G. Azzoli

**Affiliations:** 1 Thoracic Oncology Service, Division of Solid Tumor Oncology, Department of Medicine, Memorial Sloan Kettering Cancer Center, Weill Cornell Medical College, 300 E 66th Street, 12^th^ Floor, New York, NY, 10065, United States of America; 2 Sydney Medical School, University of Sydney, Sydney, NSW, 2006, Australia; 3 Division of Hematology, Oncology and Transplantation, University of Minnesota, Mayo Mail Code 480, 420 Delaware Street SE, Minneapolis, MN, 55455, United States of America; 4 Department of Epidemiology and Biostatistics, Memorial Sloan Kettering Cancer Center, 485 Lexington Avenue, New York, NY, 10017, United States of America; 5 Department of Neurology, Memorial Sloan Kettering Cancer Center, 1275 York Avenue, New York, NY, 10065, United States of America; 6 Clinical Chemistry Service, Department of Laboratory Medicine, Memorial Sloan Kettering Cancer Center, 1275 York Avenue, New York, NY, 10065, United States of America; 7 Thoracic Oncology Program, Massachusetts General Hospital Cancer Center, 55 Fruit Street, Boston, MA, 02114–2696, United States of America; Advanced Centre for Treatment, Research and Education in Cancer, Tata Memorial Center, INDIA

## Abstract

**Background:**

Lung cancers account for the majority of brain metastases which pose major therapeutic challenges. Biomarkers prognosticating for the development of brain metastases in patients with non-small cell lung cancers (NSCLC) may improve personalized care. Six serum proteomic biomarkers were previously investigated at Memorial Sloan Kettering but their associations with brain metastases were unknown.

**Methods:**

Serum NSE, CYFRA 21–1, ProGRP, SCC-Ag, TIMP1, and HE4 by ELISA-based proteomic assays were prospectively collected from consecutive patients with stage IV NSCLC. Pre-treatment serum biomarker levels as well as age, histology, and *epidermal growth factor receptor* (*EGFR*) mutation status were evaluated for association with the baseline presence of brain metastases using logistic regression and multivariable analysis. For patients without brain metastases at baseline, the cumulative incidence of subsequent brain metastases were compared according to baseline biomarkers and clinical factors using Gray’s test.

**Results:**

A total of 118 patients were enrolled, 31 (26%; 95% CI 0.19–0.35) had brain metastases at baseline and a further 26 (22%; 95% CI 0.15–0.30) developed brain metastases subsequently. Pre-treatment serum biomarker levels were available in 104 patients. There was no significant association between the six serum biomarkers and the baseline presence or subsequent development of brain metastases. Age younger than 65 years was the only clinical factor significantly associated with brain metastasis at baseline (OR 3.00; 95% CI 1.22–7.34, P = 0.02) by multivariable analysis. A trend toward increased cumulative incidence of subsequent brain metastases was observed in patients with *EGFR* mutation (p = 0.2), but this was not statistically significant possibly due to small sample size.

**Conclusions:**

Serum NSE, CYFRA 21–1, Pro-GRP, SCC-Ag, TIMP1, and HE4 are not significantly associated with brain metastases. Our methods taking into account follow-up time may be applied to independent datasets to identify a patient cohort with a higher biologic propensity for developing brain metastases. Such information may be useful for the study of agents targeting the development of brain metastases.

## Introduction

Brain metastases remain the most common form of central nervous system malignancies and approximately half of them stem from lung cancers [1, 2]. Despite advances in cancer therapy, median survival for patients with lung cancer brain metastases as a group is only 4–6 months [3]. Currently there is no approved biomarker that could be used in patients with lung cancers to reliably prognosticate for the development of brain metastases. Studies exploring the association of *epidermal growth factor receptor (EGFR)* mutation status and the development of brain metastases have yielded mixed results, and studies showing a higher incidence of brain metastases in patients with *EGFR* mutation have not taken into account the relatively longer survival of these patients [4–9]. The development of non-invasive prognostic biomarkers for brain metastases could help select high risk patients with non-small cell lung cancers (NSCLC) for more intensive brain imaging surveillance and prophylactic treatment strategies such as those proven to improve survival in small cell lung cancers [10, 11].

A previous study published in the *British Journal of Cancer* by Jacot *et al* [12] have found that high levels of serum neuron-specific enolase (NSE) may be associated with brain metastases in patients with lung cancers. The high levels of NSE was thought to be mediated by neuronal tissue damage surrounding brain metastases, however this finding was never independently validated [12]. Our group has previously published an analysis of six serum biomarkers: NSE, cytokeratin 19 fragment 21–1 (CYFRA 21–1), pro-gastrin-releasing peptide (Pro-GRP), squamous cell carcinoma antigen (SCC-Ag), tissue inhibitor of metalloproteinase-1 (TIMP1), and human epididymis protein 4 (HE4), and examined their ability to enhance non-invasive diagnosis and differentiation of histologic subtypes of lung cancers [13]. In further analysis of this dataset, we detected trends toward increased serum biomarker levels in the subset of patients with lung cancer brain metastases. We thus sought to evaluate the prognostic value of these serum biomarkers by examining their association with baseline presence and subsequent development of brain metastases in patients with NSCLC. Furthermore, we also sought to determine whether clinical factors such as age, histology, and *EGFR* mutation status, associate with the development of brain metastases, taking into account survival and follow-up time.

## Materials and Methods

### Study design and patients

This research was approved by the Memorial Sloan Kettering Cancer Center (MSK) Institutional Review Board. We conducted a prospective study at MSK with the primary objective of assessing the prognostic value of serum-based biomarkers (NSE, CYFRA 21–1, Pro-GRP, SCC-Ag, TIMP1, and HE4) [13]. Consecutive patients with metastatic lung cancers treated at MSK between 2004 and 2008 were asked to be enrolled. All patients provided written informed consent and serum samples were collected prior to the initiation of chemotherapy. The quantitative values of serum biomarkers were retrospectively analyzed for their association with the baseline presence and subsequent development of brain metastases. All patients in this analysis had pathologically confirmed stage IV NSCLC. Patient clinicopathologic characteristics including age, histology and *EGFR* mutation status were evaluated for association with the baseline presence and subsequent development of brain metastases.

### Plasma biomarker assays

Samples were collected, stored at -80°C, processed and analyzed at a MSK Clinical Laboratory Improvement Amendments (CLIA) certified laboratory. We performed serum biomarker analysis using validated commercially available Enzyme-Linked Immunosorbent Assay (ELISA) kits. The CanAg NSE EIA non-competitive immunoassay (*Fujirebio Diagnostics AB*, *Sweden*) was used with two monoclonal antibodies directed against the αγ form of the glycolytic enzyme enolase (2-phospho-D-glycerate hydrolase, EC 4.2.1.11). The CYFRA 21–1 EIA (*Fujirebio Diagnostics AB*, *Sweden*) was used with two monoclonal antibodies (MAb) specific for cytokeratin 19 in serum. The CanAg ProGRP EIA (*Fujirebio Diagnostics AB*, *Sweden)* non-competitive assay was used. The CanAg SCC EIA non-competitive immunoassay (*Fujirebio Diagnostics AB*, *Sweden*) was performed using the direct sandwich technique. The quantitative sandwich enzyme immunoassay was used to assess Human TIMP1 *(Quantikine® R&D System*, *Minneapolis*, *Minnesota)*. The HE4 EIA (*Fujirebio Diagnostics AB*, *Sweden)* was used with two mouse monoclonal antibodies (2H5 and 3D8) directed against two epitopes in the C-WFDC domain of HE4.

Ninety eight-well plates were coated and analyzed using a robotic plate analyzer. Microplates were coated with the following horseradish peroxidase-labeled MAb: anti-NSE MAb E17, anti-CYFRA 21–1 MAb, anti-ProGRP MAb E146, anti-SCC MAb, anti-TIMP1 MAb, and biotinylated anti-HE4 MAb 2H5. Serum samples were then added and incubated with the indicated monoclonal antibody. After washing, chromogen reagent (hydrogen peroxide and 3, 3´, 5, 5´ tetramethylbenzidine) was added to each well. For TIMP1, after washing an enzyme-linked polyclonal antibody specific for TIMP1 was added to the microplate. After washing, a substrate solution was added to each well.

### Statistical analysis

The levels of biomarkers were dichotomized at the upper limit of normal based on previously published data: NSE (20 ng/ml), CYFRA 21–1 (3.3 ng/ml), Pro-GRP (50 pg/ml), SCC-Ag (2.5 ng/ml), TIMP1 (58.9 μg/L), and HE4 (83 pmol/L) [14–19]. Data were obtained from a prospectively maintained anonymized clinical database at MSK.

To investigate whether these serum biomarkers (high vs. normal) and clinical factors including age, histology and *EGFR* mutation have prognostic value for brain metastasis, we first analyzed their association with presence or absence of brain metastasis at stage IV diagnosis and then with development of subsequent brain metastasis among patients who did not have baseline brain metastasis. Association of factors with presence of brain metastasis was evaluated using logistic regression. Univariate analysis was performed on all variables, and if no significant association was observed, then no further multivariable analysis was required. If a factor was found to be significantly associated with brain metastases on univariate analysis, a multivariable logistic regression model was fitted to evaluate the association adjusted for clinically relevant covariates.

Next, we captured the dates of the subsequent development of brain metastases in patients who did not have baseline brain metastasis. A time-to-event approach with competing risk methodology was used to analyze this outcome as it takes into account differences in follow-up time and the numerous deaths in this metastatic population that preclude observing a brain metastasis [20]. We used the cumulative incidence function to estimate the probability of subsequent brain metastasis where death without brain metastasis was considered a competing event. The association between biomarker levels (high vs. normal) and the cumulative incidence of subsequent brain metastasis was assessed by Gray’s test. A similar approach was used to evaluate the clinical factors for association with development of subsequent brain metastasis. For all analyses, a p-value less than 0.05 was considered significant. Competing risk analysis was analyzed using *cmprsk* package in R version 3.1.1 (http://www.R-project.org). All other statistical analysis was performed using SAS 9.4 (SAS Institute, Cary, NC)

## Results

### Patient characteristics

A total of 118 patients with stage IV NSCLC who were treatment naïve were enrolled, 57 (48%) of whom developed brain metastases, detected either at diagnosis (n = 31, 26%, 95% CI: 0.19–0.35) or subsequently during their course of treatment (n = 26, 22%, 95% CI: 0.15–0.30) (**Table 1**). The median age was 64 (range 36–85), 68 were women and 50 were men. By histologic subtyping, 99 had adenocarcinoma, 13 had squamous cell carcinoma, and 6 had NSCLC not otherwise specified. Baseline pre-treatment serum biomarkers were available for 104 patients. Attrition was due to either missed pre-treatment collection or lost samples. *EGFR* mutation testing was performed on 44 patients as testing was by clinical selection per standard of care at the time. Of those tested, 17 patients had *EGFR* mutation, of whom 11 (65%) had brain metastases either at baseline or subsequently.

**Table 1:** Distribution of clinical factors by presence of brain metastases at baseline or development of subsequent brain metastases after baseline.

		Number of Patients	Number with Brain Metastasis	
			At Baseline	After Baseline
Overall		118	31 (26%)	26 (22%)
*EGFR* mutation				
	Negative	27	5 (19%)	6 (22%)
	Positive	17	4 (24%)	7 (41%)
	Not tested	74	22 (30%)	13 (18%)
Histology				
	Adenocarcinoma	99	26 (26%)	24 (24%)
	NOS	6	2 (33%)	0 (0%)
	Squamous	13	3 (23%)	2 (15%)
				
Age	Median (Range)	64 (36–85)		
	Less than 65	61	22 (36%)	13 (21%)
	65 and older	57	9 (16%)	13 (23%)

### Association of serum-based biomarkers with brain metastasis

Of the 104 patients who had pre-treatment baseline serum biomarkers collected, there was an even distribution of patients with brain metastases at baseline (n = 26, 25%), as compared to patients who subsequently developed brain metastases (n = 25, 24%) **(Table 2)**. Median follow-up among survivors in this cohort was 7 years (range 3.6–9 years). The number of patients with baseline biomarkers high vs. normal in relation to brain metastases are detailed in **Table 2**.

**Table 2:** Distribution of serum biomarkers and other factors by presence of brain metastases at baseline or development of subsequent brain metastases after baseline.

		Number of Patients	Number with Brain Metastasis	
*Biomarker*			At Baseline	After Baseline
Overall		104	26 (25%)	25 (24%)
CYFRA 21–1				
	≤ 3.3 ng/ml	56 (54%)	12	15
	>3.3 ng/ml	48 (46%)	14	10
NSE				
	≤13 ng/ml	86 (83%)	23	19
	>13 ng/ml	18 (17%)	3	6
ProGRP				
	≤50 pg/ml	38 (37%)	12	8
	>50 pg/ml	9 (9%)	1	2
	Unknown	57 (55%)	13	15
SCCL-Ag				
	≤2.0 ng/ml	96 (92%)	25	25
	>2.0 ng/ml	8 (8%)	1	0
HE4				
	≤65 pmol/L	28 (27%)	8	3
	>65 pmol/L	76 (73%)	18	22
TIMP1				
	>55.5 μg/L	79 (76%)	21	16
	Unknown	25 (24%)	5	9

For each serum biomarker tested at baseline (NSE, CYFRA 21–1, Pro-GRP, SCC-Ag, TIMP1, and HE4), univariate analysis of individual biomarkers did not yield any association with the presence of brain metastasis at baseline thus no further multivariable analysis was performed (**Table 3**).

**Table 3:** Univariate logistic regression analysis for association of biomarkers with presence of brain metastasis at baseline.

*Biomarkers*		N	OR (95%CI)	p-value
CYFRA 21–1				
	≤ 3.3 ng/ml	56	1.0	
	>3.3 ng/ml	48	1.51 (0.62,3.68)	0.37
NSE				
	≤13 ng/ml	86	1.0	
	>13 ng/ml	18	0.55 (0.15,2.07)	0.38
ProGRP				
	≤50 pg/ml	38	1.0	
	>50 pg/ml	9	0.27 (0.03,2.42)	0.24
SCCL-Ag				
	≤2.0 ng/ml	96	1.0	
	>2.0 ng/ml	8	0.41 (0.05,3.46)	0.41
HE4				
	≤65 pmol/L	28	1.0	
	>65 pmol/L	76	0.78 (0.29,2.06)	0.61
				

**Fig 1:**
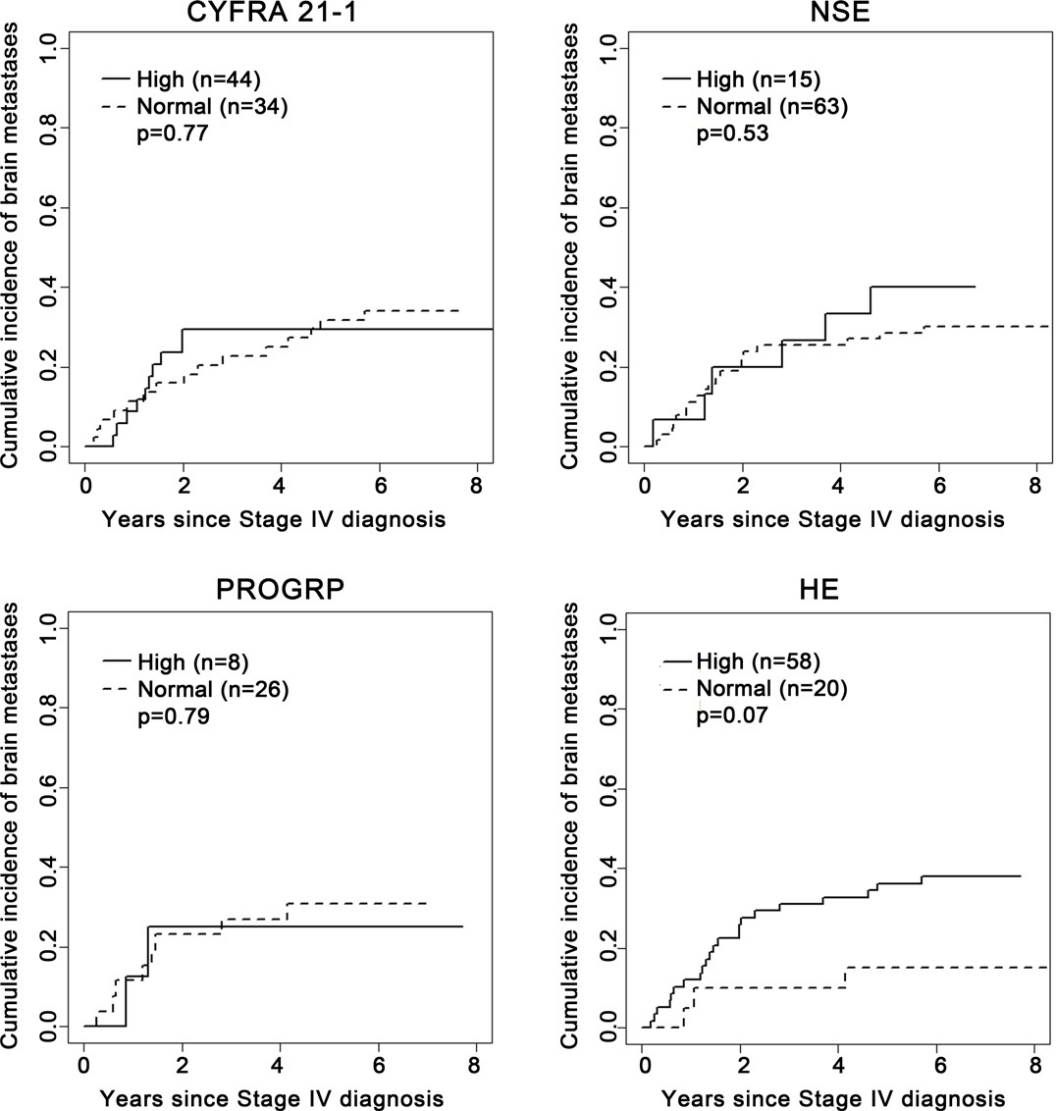
Association of serum biomarkers with cumulative incidence of subsequent brain metastasis in patients who did not experience brain metastasis at baseline.

In patients without brain metastasis at baseline, there were no significant differences between patients with high vs. normal pre-treatment biomarker levels in relation to the incidence of subsequent brain metastases across all time points (**Fig 1**). There was a trend toward higher cumulative incidence of brain metastases among patients with high baseline HE4 compared to those with normal levels of HE4 but this was not statistically significant (P = 0.07). No further multivariable analysis was performed since no factors were significantly associated with the cumulative incidence of subsequent brain metastasis. Statistical comparisons of subsequent brain metastases based on TIMP1 and SCC-Ag levels were not possible due to too few patients or events (**Table 2**).

### Association of age and NSCLC histology with brain metastases

**Fig 2:**
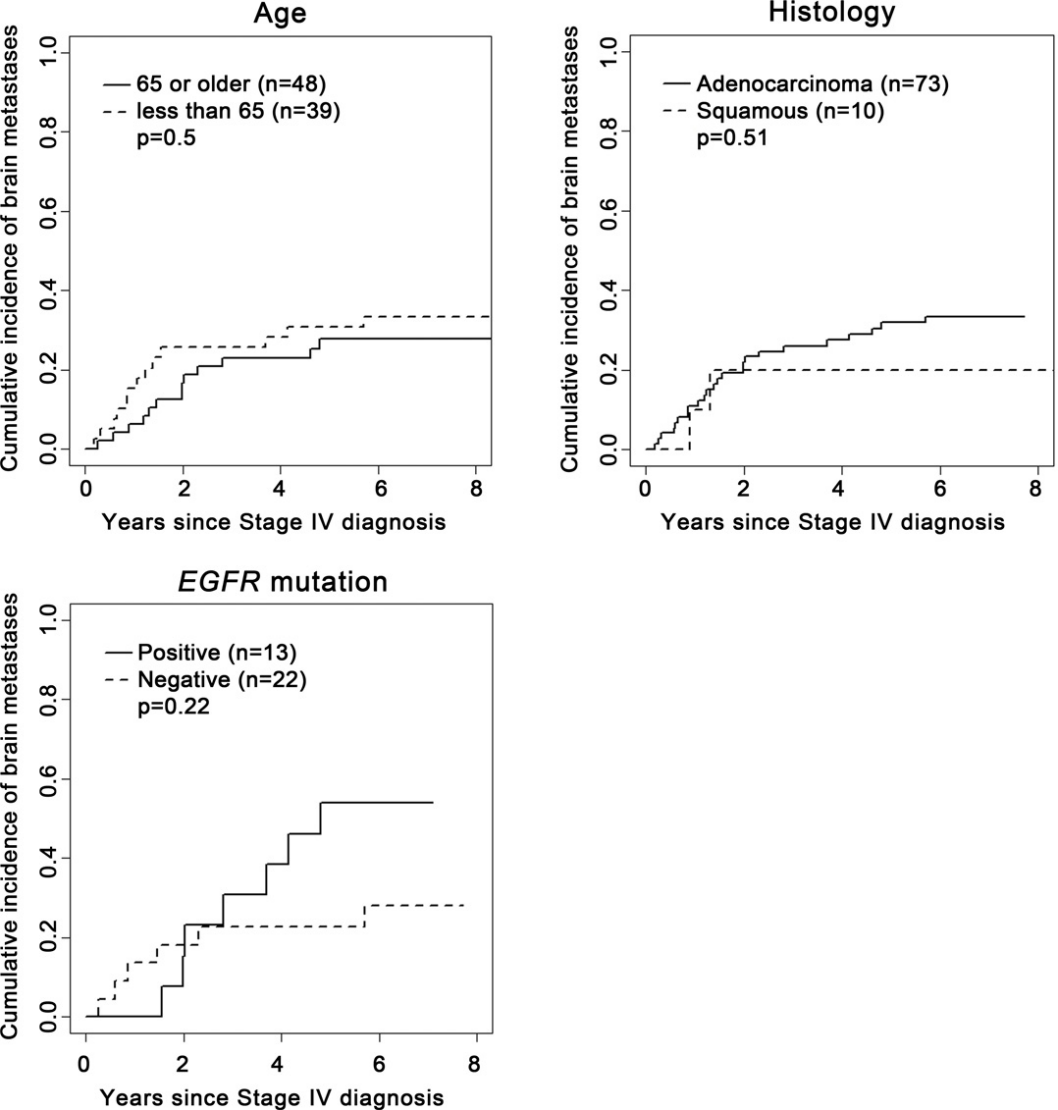
Association of clinical factors with cumulative incidence of subsequent brain metastasis in patients who did not experience brain metastasis at baseline.

Compared to patients aged 65 years or older, patients younger than 65 years had significantly more brain metastases at baseline (36% vs 16%; Odds Ratio [OR 3.01], 95% CI: 1.24–7.28, P = 0.01) **(Table 3)**. This association remained significant in multivariable analysis adjusting for histology and *EGFR* mutation status (OR 3.00, 95% CI: 1.22–7.34, P = 0.02) **(Table 4)**. However, among patients without baseline brain metastases, there was no significant difference between the age groups in the incidence of subsequent brain metastases (P = 0.5) **(Fig 2)**.

**Table 4:** Univariate and multivariable logistic regression analysis for association of clinical factors with presence of brain metastases at baseline.

			Univariate Analysis		Multivariable Analysis**	
Clinical Factors		N	OR (95%CI)	p-value	OR (95%CI)	p-value
*EGFR* mutation*						
	Negative	27	1.0		1.0	
	Positive	17	1.35 (0.31,5.96)	0.69	1.30 (0.29,5.94)	0.73
Histology*						
	Adenocarcinoma	99	1.0		1.0	
	Squamous	13	0.84 (0.22,3.30)	0.81	0.77 (0.18,3.30)	0.73
Age						
	65 and older	61	1.0		1.0	
	Less than 65	57	3.01 (1.24, 7.28)	0.01	3.00 (1.22, 7.34)	0.02

* All patients (N = 118) were included in univariate and multivariable analysis but odds ratios for NOS histology and unknown *EGFR* mutation are not shown

** Multivariable model includes *EGFR* mutation status, histology and age.

With regard to histology, there were no significant differences between patients with adenocarcinoma and squamous cell carcinoma histology in relation to the baseline presence of brain metastasis (OR = 0.84, 95% CI: 0.22–3.30, P = 0.81) (**Table 3**). In patients without brain metastasis at baseline, there was no significant difference between patients with adenocarcinoma and squamous cell carcinoma histology in relation to their cumulative incidence of subsequent brain metastases across all time points (P = 0.51) (**Fig 2**).

### Association of *EGFR* mutation status with lung cancer brain metastasis

Of the 44 patients tested for *EGFR* mutation, 17 patients were tested positive, of whom 11 (65%) had brain metastases by the end of the study, including 4 at baseline and 7 subsequently. In comparison, only 11 of 27 patients (41%) tested negative for *EGFR* mutation had brain metastases by the end of the study, including 5 at baseline and 6 subsequently (**Table 1**). The OR associating *EGFR* mutation with baseline brain metastases was 1.35 (P = 0.69) (**Table 2**). In patients without brain metastases at diagnosis of stage IV NSCLC (n = 35), there was a trend toward higher cumulative incidence of brain metastases among patients tested positive for *EGFR* mutation compared to those tested negative. (P = 0.22) (**Fig 2**).

## Discussion

Our study did not find a significant association between any of 6 pre-treatment serum biomarkers and the baseline presence or the subsequent development of brain metastases in patients with stage IV NSCLC. Our study refuted the hypothesis by Jacot *et al* [12] that serum NSE may be a specific marker for neuronal damage from brain metastases. Methodological differences may have accounted for the differences in conclusions, since the study by Jacot *et al* [12] examined the survival of patients with lung cancer brain metastases, and our study examined all metastatic lung cancer patients for their baseline or subsequent development of brain metastases. An older study of patients with small cell lung cancers by van de Pol *et al* [21] also showed that while serum NSE levels rose with the development of metachronous brain metastases, changes in NSE levels were not specific to intracranial disease activity. While our published primary analysis [13] did confirm the finding by Jacot *et al* [12] that baseline pre-treatment NSE level was prognostic for overall survival (HR 1.266, P = 0.0298), our study suggests that serum NSE is not a specific biomarker for lung cancer brain metastases and such further studies should not be pursued.

To date, no serum biomarker for brain metastases has been validated in patients with NSCLC. Lee et al [22] found that pre-treatment serum carcinoembryonic antigen correlated with brain metastases in patients with NSCLC. However, an independent validation study would need to take into account timing of brain metastases and survival for a biomarker to be deemed biologically relevant and clinically useful in selecting patients at high risk for subsequent development of brain metastasis for personalized care.

The observation from this study that patients with age younger than 65 years are significantly associated with brain metastases at baseline is consistent with previously published reports [11, 23]. A large retrospective review of the Southwest Oncology Group (SWOG) database by Gaspar et al [23] revealed that younger age and adenocarcinoma histology were associated with the development of brain metastases. Whether younger age and adenocarcinoma histology have a higher biologic propensity toward the development of brain metastases could not be adequately addressed by this study given the small number of patients that developed subsequent brain metastasis (25 events). Any further attempts at investigating the association of age and histology with the development of brain metastases using independent datasets needs to take into account differences in survival and follow-up time.

Due to the limited number of patients who had undergone *EGFR* mutation at the time of this study, we were not able to conclusively solve the controversy regarding the association between *EGFR* mutation and the development of brain metastases. However, our methods were substantially different to previous studies in attempting to address this question [4–9]. Many previous studies have looked at a set of patients with NSCLC and known brain metastases, and looked at the incidence of *EGFR* mutations within this selected group without accounting for survival and time to development of brain metastases [4, 5, 7]. While several reports have found a higher incidence of brain metastases in patients with *EGFR* mutant lung cancers, it has never been shown whether this is due to a biologic propensity or simply a result of the longer survival of these patients due to EGFR targeted therapy [24–26]. In our study, we have not only assessed for the presence of brain metastases, but also time to the development of brain metastases across all time points thus taking into account differences in survival and follow-up time.

There are several limitations in this study. The analysis was done retrospectively which may have introduced bias. Furthermore, the sample size was relatively small and the limited number of *EGFR* mutation testing have prevented any firm exploratory statistical analysis. Attrition due to missed collections or lost samples highlights the practical challenges in designing a biomarker study. Despite these limitations and negative results, there was no evidence to suggest that the serum biomarkers studied can be used clinically to prognosticate for the development of brain metastases. While there was a trend toward an association between high baseline HE4 and increased subsequent development of brain metastases (p = 0.07), such a biomarker cannot be recommended for clinical use without independent validation of a highly significant association. Given serum HE4 was recently shown to be associated with poor prognosis in patients with NSCLC [27], further validation studies may be justified.

The main strength of this study is our methods in determining the association of biomarkers with brain metastases both uniformly at baseline and subsequently, and accounting for time and patient survival. In order to confirm the association between *EGFR* mutation or other biomarkers and the development of brain metastases over time, independent datasets may be analyzed using the same methods.

It is worth pointing out that while non-invasive prognostic biomarkers for the development of brain metastases are important for improving personalized therapies of patients, such biomarkers must be shown to be highly sensitive and specific in order to be clinically useful. Any positive association found by exploratory analyses must be independently validated before clinical application [28]. Using serum NSE as an example, despite earlier reports, our independent study found that it is not a specific biomarker for lung cancer brain metastases. Furthermore, large independent datasets do not exist for all biomarkers. Thus when developing novel serum biomarkers for brain metastases, a high magnitude of effect is essential for their potential clinical utility.

In conclusion, this independent biomarker study found that the 6 pre-treatment serum biomarkers including NSE were not associated with the baseline presence or subsequent development of brain metastases in patients with metastatic lung cancers. Our methods may be applied to independent datasets to identify a patient cohort with a higher biologic propensity for developing brain metastases taking into account difference in follow-up time. Such information may be useful for the study of agents targeting the development of brain metastases.
