# Correction: Serum Biomarkers Associated with Clinical Outcomes Fail to Predict Brain Metastases in Patients with Stage IV Non-Small Cell Lung Cancers

**DOI:** 10.1371/journal.pone.0152450

**Published:** 2016-03-23

**Authors:** The PLOS ONE Staff

## Notice of Republication

The original Supporting Information files were published in error. The files were removed from the HTML and PDF versions of this article on March 11, 2016. The publisher apologizes for the error.
